# Adverse childhood experiences, associated stressors and comorbidities in children and youth with fetal alcohol spectrum disorder across the justice and child protection settings in Western Australia

**DOI:** 10.1186/s12887-022-03654-y

**Published:** 2022-10-10

**Authors:** Grace Kuen Yee Tan, Martyn Symons, James Fitzpatrick, Sophia G. Connor, Donna Cross, Carmela F. Pestell

**Affiliations:** 1 School of Psychological Science (M304), The University of Western Australia, 35 Stirling Highway, Crawley, Western Australia 6009 Australia; 2 Patches Australia, Subiaco, Australia; 3 Telethon Kids Institute (TKI), Nedlands, Australia; 4 Royal Perth Hospital, Perth, Australia

**Keywords:** Fetal alcohol Spectrum disorder, Neglect, Trauma, Adverse childhood experiences, Comorbidities, Child protection system, Justice system

## Abstract

**Background:**

Individuals with Fetal Alcohol Spectrum Disorder (FASD) are at risk of having adverse childhood experiences (ACEs), especially those with child protection and/or justice system involvement. The complex relationship between FASD and psychosocial vulnerabilities in the affected individual is an important clinical risk factor for comorbidity. This study (1) explored the ACEs and associated stressors in individuals with FASD; (2) investigated the association between ACEs and negative outcomes, i.e., justice/child protection system involvement; and (3) examined the relationship between ACEs and comorbid conditions such as mood and neurodevelopmental disorders.

**Methods:**

Data were collected retrospectively via file review from diagnostic clinics in Western Australia. Life adversity was coded using a standardised ACEs questionnaire. A total of 211 participants (72% males) with FASD with a mean age of 11 years (range = 2–21) were included in the final sample. 70% of the total sample had been involved with the child protection system and 40% had trouble with the law.

**Results:**

Exposure to drinking/substance misuse at home (70%) and domestic violence (52%) were the two most common ACEs across the total sample. In the entire cohort, 39% had four or more ACEs, indicating higher risks of poor health outcomes. Additional stressors recorded were disengagement from school (43%), transiency (19%), victims of bullying (12%), traumatic brain injury (9%) and homelessness (5%). ACEs such as drinking/substance misuse at home, emotional neglect and physical neglect were positively associated with child protection system involvement. Additionally, exposure to domestic violence was positively correlated with justice system involvement. Higher rates of life adversity in this clinical population were associated with an increased number of comorbidities. Specifically, those with FASD who had comorbidities such as attachment disorder, substance use disorder, and PTSD also reported higher ACEs scores.

**Conclusion:**

ACEs were common in this clinical population. Increased ACEs in this sample were associated with increased comorbidities and involvement with the child protection and/or justice system. This highlights that prevention, intervention and early diagnosis of FASD are important for at risk children to reduce the negative effects of ACEs.

**Supplementary Information:**

The online version contains supplementary material available at 10.1186/s12887-022-03654-y.

## Background

Alcohol is a teratogen, a substance that can alter growth and normal development in the central nervous system, including brain structure and other organs of a developing foetus [1]. Fetal Alcohol Spectrum Disorder (FASD) is a condition characterised by a variety of cognitive, physical, emotional and behavioural difficulties resulting from prenatal alcohol exposure (PAE). A meta-analysis estimated the worldwide prevalence of FASD to be 0 to 176.7 per 1000 births [2]. The prevalence rate of FASD in Australia is mainly monitored through passive case ascertainment; thus, the figures are likely to be an underestimate [3]. In Western Australia (WA), the estimate in 2015 was 0.26 per 1000 births [4]. While FASD is a condition that can potentially affect individuals from all socioeconomic and cultural backgrounds [5, 6], some communities are more at risk. The rates of FASD in a group of remote Australian Aboriginal communities was the highest, with a reported prevalence of 194 per 1000 births [7]. The causes of excessive drinking in Aboriginal communities are multifaceted and are a product of cultural dislocation due to colonisation, intergenerational trauma and social/economic marginalisation [8, 9].

Children born with FASD often encounter a range of adverse psychosocial situations, such as having early childhood characterised by parental unemployment and substance/alcohol misuse, mental health problems, and child protection involvement [10, 11]. In Australian Aboriginal children with FASD, these adverse environmental factors may also occur within the context of historical and intergenerational trauma [12]. Price et al. proposed that when a child with FASD is born into a dysfunctional family characterised by substance misuse, family violence and lacking empathy/communication, the functional impairments as a result of PAE are likely to be exacerbated [13]. Consequently, the expression of FASD could be characterised by a series of negative events starting from the initial PAE to the accumulation of adverse childhood experiences (ACEs) over the lifespan [11]. ACEs are traumatic events that range from abuse (e.g., sexual, physical and emotional), neglect (e.g., emotional and physical) to household dysfunctions (e.g., parental separation/divorce, parental mental illness, domestic violence, household substance misuse, incarceration) [14].

Importantly, individuals who endorsed four or more categories of ACEs were 4 to 12 times more likely to engage in health-risk behaviours (e.g., alcohol/substance misuse, suicide attempt) and have chronic health problems (e.g., cancer, coronary heart disease) in adulthood; while those with one to three ACEs did not fare as well as those who had experienced none [14]. In the context of FASD, pregnant women who reported a higher number of ACEs were more likely to consume alcohol to cope with stress and anxiety associated with ongoing life stressors [15]. In addition to the 10 categories of ACEs outlined above, individuals with FASD are also at an increased risk of experiencing other stressors in life such as coming into contact with the child protection and justice system [16, 17]. For example, a recent study reported the prevalence rate of FASD among young people in an Australian youth detention centre as 36% [18].

Further, the complex relationship between FASD and psychosocial vulnerabilities in the affected individual is also an important clinical risk factor for comorbidity [19]. It is well-established that individuals with FASD are at risk of developing a range of comorbid disorders [20]. For example, Weyrauch et al. found that they are 11 times more likely to experience an anxiety disorder and 10 times more likely to be diagnosed with attention-deficit-hyperactivity disorder (ADHD) than the general population [21]. Other frequently occurring comorbidities include disorders of the nervous system, conduct disorder, receptive/expressive language disorders, hearing impairment and intellectual disabilities [20]. In an Australian FASD sample, Connor et al. found ADHD, sleep disturbance and anxiety disorder were the three most common comorbidities reported [5].

To date, a handful of studies [13, 22] have explored the relationship between PAE and early life trauma, with four published papers using a standardised ACEs questionnaire in the FASD population [11, 23–25]. These studies revealed high rates of early life adversity in individuals with FASD. Additionally, it was also documented that childhood trauma is associated with child protection or justice system involvement, especially among children with FASD [11, 26]. However, little to no research has examined the ACEs profiles of those with FASD who have been in contact with these government systems. To our knowledge, this study is the first to explore ACEs in an Australian FASD sample characterised by a high proportion of individuals who had been involved with the child protection and/or justice systems. Notably, both (i.e., child protection/justice) represent priority populations for the reduction of ACEs given their psychosocial vulnerabilities. A better understanding of the frequency and type of ACEs in at-risk children is important to help improve their physical, social/emotional and behavioural outcomes through the development of better early screening tools, services and more targeted interventions [27]. Consequently, this study aimed to (1) explore the ACEs and associated stressors in children and youth with FASD; (2) investigate the association between ACEs and negative outcomes, i.e., justice/child protection system involvement; (3) and examine the relationship between ACEs and comorbid conditions in the sample.

## Method

### Participants

Between November 2016 and June 2019, 480 individuals attended Patches Australia, a multidisciplinary FASD diagnostic assessment service operating across Western Australia (WA). A total of 226 participants met the Australian FASD diagnostic criteria [28] and 254 individuals were not diagnosed with FASD. Participants’ files were reviewed retrospectively, and individuals were included if they had a diagnosis of FASD and were under 22 years of age. The age cut-off was determined using the median absolute deviation method. Specifically, young people with FASD who were above the age of 21 were excluded from the present study (*n* = 15). This resulted in a final sample size of 211 individuals (151 males) with FASD aged 2 to 21 years old.

### Diagnostic process

In the Patches multidisciplinary FASD clinics, referrals for the diagnostic assessment came from general practitioners, paediatricians, caseworkers and other health, justice or education service providers. A paediatrician and a neuropsychologist were always part of the team, while a speech pathologist was only usually present when the participant was a child/adolescent. Participants were diagnosed with FASD using the Australian FASD diagnostic guidelines [28]. As part of the FASD diagnostic process, participants’ neurocognitive performances were assessed in multiple domains including executive functioning, cognition, memory, attention, academic achievement, language, motor, affect regulation and adaptive functioning. Participants’ medical, psychosocial and developmental history was gathered through clinical interviews with the client and/or parents/legal guardians and self-report/informant questionnaires.

### Adverse Childhood Experiences (ACEs) and associated stressors

Psychologists or paediatricians on the Patches multidisciplinary team routinely gather information on early life adversities through a clinical interview with the carer/parent and the child which is then included in the FASD diagnostic reports. For example, when a child was suspected of having experienced sexual abuse, the topic was approached delicately by an experienced clinician on the diagnostic team during the clinical interview with the child. These reports and all other available source documents (e.g., allied health, medical and educational reports) were reviewed, and the presence of nine early life adversities (e.g., victim of physical abuse, sexual abuse, emotional/verbal abuse, physical neglect, emotional neglect) and household dysfunctions (e.g., exposure to domestic violence, drinking/substance misuse, incarcerated relative, mentally ill family members/family members who attempt suicide) were retrospectively coded by the researcher against the 10-item ACEs questionnaire (See Supplementary Material) [14]. No missing information apart from participants’ parental marital status was identified during the retrospective coding of data. This left only nine categories comprising the ACEs total score. Therefore, a maximum score of nine (range = 0–9) was attainable. A score of four or more indicated an increased risk of negative health outcomes [14, 29]. The ACEs questionnaire has acceptable (i.e., 0.70 or higher) internal reliability consistency and test-retest reliability [29, 30]. A satisfactory convergent validity with the Childhood Trauma Questionnaire has also been demonstrated in both clinical and non-clinical samples [31]. Furthermore, the ACEs questionnaire has been widely used to capture childhood adversity in both adults [14, 32] and paediatric samples (e.g., FASD, autism spectrum disorder, at-risk children) [11, 24, 33].

Additional life stressors (i.e., involvement with child protection, justice systems, victims of bullying, homelessness, transiency, severe traumatic brain injury, disengagement from school) not included in the 10-item ACEs questionnaire were also gathered from the Patches team diagnostic reports.

### Diagnosis of comorbid conditions

Information on participants’ pre-existing diagnoses of neurodevelopmental, medical or mental health conditions was gathered from allied health and medical reports. Additional diagnoses were given by the Patches diagnostic team if criteria according to the Diagnostic and Statistical Manual of Mental Disorders (DSM-5) were met [34]. Only comorbidities with a frequency of at least 10% were coded as binary variables (Yes/No) and included in the analysis. These included ADHD, sleep disorder, attachment disorder, anxiety disorder, hearing impairment, post-traumatic stress disorder (PTSD), intellectual disability (ID), substance use disorder, conduct disorder and depression.

### Ethics and consent

This study was approved by the Human Ethics Committee from the University of Western Australia and the Western Australian Aboriginal Health Ethics Committee (HREC Approval Number: 901) in consultation with an Aboriginal community reference group. For participants who were over 18 and capable of providing informed consent, consent was obtained in writing or electronically from the participant at the time of assessment. For participants under 18, or who were 18 or older and not capable of providing informed consent, assent was obtained from the participant and consent was obtained in writing or electronically from each participant’s parent or legal guardian at the time of assessment. Consent/assent was provided by all participants for their data to be included in this study.

### Statistical analysis

Data analysis was conducted using IBM SPSS-22. Descriptive analyses were conducted to examine the demographics of the total sample, demographics of the subgroups (i.e., child protection engagement, justice system involvement) as well as the frequency and proportion of ACEs scores/categories and associated stressors.

An alpha level of 0.05 was used for all statistical tests. Pearson’s correlation was employed to explore the relationship between the total ACEs scores of the overall sample and age. Point-biserial correlations were used to examine the association between total ACEs scores of the entire cohort and demographic variables such as sex (1 = Male, 0 = Female) and cultural background (1 = Australian Aboriginal, 0 = Caucasian). The relationship between child protection system involvement (1 = Yes, 0 = No involvement) and total ACEs scores was also explored using point-biserial correlations. Similarly, the same test was used to examine the relationship between justice system involvement (1 = Yes, 0 = No) and total ACEs scores. Only participants aged 10 and above were included in this test as the age of criminal responsibility in WA is 10 years old.

A series of logistic regressions were performed to investigate the predictors (age, sex, cultural backgrounds) of each ACEs category in the total sample. All assumptions for the regression analyses were checked and met unless otherwise stated. The relationships between each ACEs category and child protection system involvement were explored using a series of Phi coefficients. The associations between each category of ACEs and justice system involvement were also examined with Phi coefficients in individuals aged 10 and above.

Descriptive statistics were used to explore the mean ACEs scores and standard deviation for each stressor and comorbid condition. Pearson’s correlation was performed to investigate the relationship between the total ACEs scores and the total number of comorbidities across the sample. This was followed up by a series of point-biserial correlations to explore the relationship between the total ACEs scores and each comorbid condition. The Benjamini-Hochberg procedure [35] was used to correct for multiple comparisons for each family of tests.

## Results

### Participant Demographics

The mean age of the total sample at the time of assessment was 11 years (SD = 5, range = 2–21). The majority were individuals aged below 18 (*n* = 199, 94%). Most participants were males (*n* = 151, 72%) and identified as Australian Aboriginal (*n* = 163, 77%). Of the total sample, 137 (64.9%) came from regional or remote parts of WA, while 74 (35%) were from major cities. Across the entire sample, 147 (70%) had contact with the child protection system, and 85 (40%) were involved with the justice system. Demographic characteristics based on child protection/justice system involvement can be seen in Table 1.

**Table 1:** Participant demographics based on child protection/justice system involvement

Demographics	Child Protection System Involvement	Justice System Involvement
	*n* = 147 (%)	*n* = 85 (%)
Mean Age (SD)	11 (4)	15 (2)
Sex		
Male	102 (69)	72 (85)
Female	45 (31)	13 (15)
Cultural Background		
Aboriginal	113 (77)	73 (86)
Caucasian	34 (23)	12 (14)
Geographical Area		
Major cities	54 (37)	32 (38)
Regional/remote	93 (63)	53 (62)

### Adverse Childhood Experiences (ACEs) Scores

The mean ACEs scores for the entire cohort were 2.8 (SD = 1.9, range = 0–8). In the overall sample, 83 (39%) had four or more ACEs recorded (See Table 2). The total ACEs scores of the overall sample were significantly positively correlated with age, r(211) = .14, *p =* .04. Specifically, there were more documented ACEs in the records of older children than younger children. However, there was no association between the total ACEs scores and sex, r(211) = .06, *p =* .36. Similarly, no significant correlation was found between the total ACEs scores and cultural background, r(211) = .03, *p =* .71. Point biserial correlation shows young people with FASD who had been involved with the child protection system had higher total ACEs scores, r(211) = .38, *p* < .001. Similarly, this trend was also observed in those with justice system involvement, r(133) = .17, *p* = .047.

**Table 2:** Frequency and proportion of nine categories of adverse childhood experiences (ACEs) in the overall sample and by groups

ACEs Categories	Overall Sample	Child Protection System Involvement	Justice System Involvement
	*N* = 211 (%)	*n* = 147 (%)	*n* = 85 (%)
Drinking/substance misuse at home	148 (70)	117 (80)	65 (77)
Domestic Violence	109 (52)	83 (57)	58 (68)
Physical Neglect	98 (46)	86 (59)	38 (45)
Emotional Neglect	97 (46)	86 (59)	37 (44)
Physical Abuse	42 (20)	35 (24)	18 (21)
Parental Incarceration	38 (18)	31 (21)	21 (25)
Emotional/Verbal Abuse	23 (11)	18 (12)	9 (11)
Suicide attempt/mentally ill family	22 (10)	12 (8)	15 (18)
Sexual Abuse	22 (10)	18 (12)	13 (15)
Total ACEs Scores			
Zero	31 (15)	10 (7)	5 (6)
One to three	97 (46)	66 (45)	42 (49)
Four or more	83 (39)	71 (48)	38 (45)

### ACEs Categories in the Total sample

The most common ACEs in the entire sample was exposure to drinking/substance misuse at home (70%). Other common ACEs included domestic violence (52%), physical neglect (46%), and emotional neglect (46%) – (Table 2). In the overall sample, logistic regression results (Table 3) show that age and being a male were associated with an increased risk of exposure to domestic violence, being a victim of sexual abuse and having a family member who was mentally ill or had attempted suicide. However, these results were no longer significant when the Benjamini-Hochberg corrections were applied.

**Table 3:** Logistic regressions predicting adverse childhood experiences (ACEs) categories from demographic variables in the overall sample

ACEs Categories	Odds Ratio (95% CI)	Odds Ratio (95% CI)	Odds Ratio (95% CI)
	Age	Male^b^	Cultural background^b^
*Model 1*			
Drinking/Substance misuse at home	1.02 (.95, 1.09)	1.30 (.67, 2.53)	1.35 (.68, 2.69)
*Model 2*			
Domestic Violence	1.09^a^ (1.02, 1.17)	2.37^a^ (1.24, 4.53)	1.41 (.71, 2.78)
*Model 3*			
Emotional Neglect	1.00 (.94, 1.06)	.73 (.39, 1.36)	.92 (.48, 1.76)
*Model 4*			
Physical Neglect	1.00 (.94, 1.07)	.74 (.40, 1.38)	.94 (.49, 1.80)
*Model 5*			
Physical Abuse	1.01 (.93, 1.09)	1.38 (.61, 3.14)	.49 (.23, 1.04)
*Model 6*			
Parental Incarceration	1.02 (.94, 1.10)	1.51 (.64, 3.60)	1.67 (.65, 4.26)
*Model 7*			
Suicide attempt/mentally ill family members	1.14^a^ (1.02, 1.27)	1.41 (.44, 4.50)	1.91 (.52, 6.88)
*Model 8*			
Sexual Abuse	1.15^a^ (1.03, 1.28)	.37^a^ (.14, .98)	1.29 (.41, 4.10)
*Model 9*			
Emotional/Verbal Abuse	1.02 (.92, 1.12)	.88 (.33, 2.34)	.63 (.24, 1.65)

^a^results were no longer significant after the Benjamini-Hochberg correction for multiple comparisons was applied

^b^Reference Group = females and Caucasians

### ACEs Categories and Child Protection System Involvement

Phi coefficient tests show that drinking/substance misuse at home (Φ = .31, *p* < .001, Hochberg threshold = .005), emotional neglect (Φ = .38, *p* < .001, Hochberg threshold = .005) and physical neglect (Φ = .37, *p* < .001, Hochberg threshold = .005) were positively associated with child protection system involvement. These associations remained significant even after applying the Benjamini-Hochberg corrections. However, domestic violence (Φ = .31, *p* = .034, Hochberg threshold = .015) and physical abuse (Φ = .15, *p* = .031, Hochberg threshold = .015) were no longer significant once corrections for multiple comparisons were applied. ACEs including parental incarceration (*p* = .078), suicide attempt/mentally ill family members (*p* = .104), sexual abuse (*p* = .192) and emotional abuse (*p* = .345) were not associated with child protection system involvement.

### ACEs Categories and Justice System Involvement

For those aged 10 and above, documented exposure to domestic violence was positively associated with justice system involvement (Φ = .28, *p* = .001, Hochberg threshold = .005). However, the relationship between parental incarceration and justice system involvement was no longer significant once corrections were applied (Φ = .17, *p* = .046, Hochberg threshold = .010). Drinking/substance misuse at home (*p* = .051), suicide attempt/mentally ill family members (*p* = .066), sexual abuse (*p* = .434), emotional neglect (*p* = .476), physical neglect (*p* = .560), emotional abuse (*p* = .740) and physical abuse (*p* = .741) were not significantly associated with justice system involvement.

### Associated Stressors

Other stressors in life not measured by the ACEs questionnaire were reported in Table 4. Almost half (43%) of the total sample disengaged from school. Other less common stressors recorded were transiency, documented victims of bullying, sustained a traumatic brain injury and homelessness.

**Table 4:** Frequency of associated stressors in the overall sample and the corresponding mean adverse childhood experiences (ACEs)

Associated Stressors	*N* = 211 (%)	Mean ACEs (SD)
Disengagement from school	91 (43)	3.3 (1.8)
Transiency	40 (19)	3.3 (1.8)
Documented victims of bullying	26 (12)	2.5 (1.9)
Sustained severe traumatic brain injury	19 (9)	3.2 (1.3)
Homelessness	11 (5)	3.8 (1.7)

### Comorbid Conditions

The total number of comorbid conditions across all participants ranged from 0 to 8 (mean = 2.3, SD = 1.7) – See Table 5. Higher total ACEs scores in the overall sample were associated with an increased number of comorbidities, r(211) = .27, *p <* .001. Specifically, those who had comorbidities such as substance use disorder r(211) = .19, *p =* .006, Hochberg threshold = .015, attachment disorder r(211) = .24, *p* = .001, Hochberg threshold = .010, and PTSD r(211) = .26, *p <* .001, Hochberg threshold = .005, also had higher ACEs scores. Conversely, individuals with FASD who also had ID reported lower ACEs scores, r(211) = −.17, *p* = .012, Hochberg threshold = .020. These correlations remained statistically significant even after the Benjamini-Hochberg corrections were applied.

**Table 5:** Mean adverse childhood experiences (ACEs) for the total sample by different comorbidities

10 Most Common Comorbidities	*N* = 211 (%)	Mean ACEs (SD)
ADHD	89 (39)	3.0 (2.0)
Sleep disorder	77 (34)	3.0 (1.9)
Attachment disorder	65 (29)	3.7 (1.7)
Anxiety disorder	61 (27)	3.2 (1.9)
Hearing impairment	57 (25)	2.9 (2.0)
Post-traumatic stress disorder	55 (24)	3.9 (1.7)
Intellectual disability	48 (21)	2.3 (1.8)
Substance use disorder	35 (15)	3.6 (1.8)
Conduct disorder	27 (12)	2.9 (1.8)
Depression	27 (12)	3.5 (1.8)
0 to 2 diagnoses	127 (60)	2.4 (1.8)
3 to 5 diagnoses	76 (36)	3.3 (1.8)
6 to 8 diagnoses	8 (4)	4.5 (1.6)

## Discussion

To the best of our knowledge, this is the first Australian study to explore the types and frequency of ACEs and associated stressors in children and youth with FASD. This study highlighted that ACEs were common in this clinical population, particularly those involved with the child protection and/or justice system.

### ACEs in the overall FASD Sample

The highest frequency of ACEs documented in the overall sample was exposure to drinking/substance misuse at home. This was present in 70% of the cases and aligned with results from a Canadian FASD/PAE sample (70%) [24]. Alcohol/substance misuse was previously found to be associated with social and economic disadvantage, especially in parents of children with FASD [6]. This highlights the importance of multilevel interventions that address the economic/social disparities among marginalised populations to reduce the negative effects of alcohol/substance misuse. Additionally, given past research [36, 37] shows that child maltreatment and domestic violence are commonly associated with alcohol/substance misuse, this again emphasises the need for multidimensional interventions to support families and children living with FASD.

Domestic violence was present in half of this sample. This may be due to the high number of participants in the current study from regional/remote areas, where domestic violence is more common as a result of unique geographical and social structures in these communities [38]. Two other ACEs categories, including emotional neglect and physical neglect were experienced by approximately 46% of the overall sample, rates (i.e., 40%) that were similar to a vulnerable/disadvantaged paediatric population from South Western Sydney [39]. Additionally, the rate of reported sexual abuse in this sample was higher (11% in our study versus 8%) than the national statistics of substantiated sexual abuse in children involved in the Australian child protection system [40]. While the rate of sexual abuse seems to be higher in our study, this may still be an underestimate as it is common for children to not disclose sexual abuse due to shame/fear, a lack of opportunity, concerns about consequences to others/self and inappropriately being accustomed to the abuse incidents [41, 42]. Similarly, 10% of the overall sample had a family member who was mentally ill or had previously attempted suicide, rates that were nearly twice as high as the 6% base rate of suicide in Aboriginal and Torres Strait Islander Peoples and 2% in non-Aboriginal people in WA [43]. For children with FASD, instability in their caregiving environment can have significant negative consequences on their socioemotional development and educational engagement [44].

Findings from this study build upon evidence related to ACEs profiles in individuals with FASD in countries other than Australia. Similarly, an American study [11] found ACEs tend to be more prevalent in individuals with FASD who had been involved with the child protection system. Interestingly, the mean ACEs score was higher (5.3 versus 2.8 in our sample) in the study by Kambeitz et al. [11]. This may be because the North Dakota FASD Centre is a tertiary referral centre where most of the referrals were FASD cases with complex backgrounds and severe clinical presentations. Further, the total ACEs scores were summed from 10 items, while the current study only had complete data for nine ACEs items. Thus, this difference may explain the higher rates of ACEs in the American FASD group. Similarly, children with FASD/PAE from a Canadian sample [24] also reported higher mean ACEs scores (3.4 compared with 2.8 in our sample). This may be because a different version of the ACEs questionnaire was employed in the study; thus, capturing somewhat different types of life adversity (e.g., 97% of participants were recorded as “not raised by both biological parents”).

Arguably, an important finding in the current study was that 39% of the total sample had four or more ACEs categories documented across their lifespan, rates (26 to 31%) which were much higher than individuals with other physical/neurodevelopmental disabilities (e.g., hearing/visual disability, intellectual disability) [45, 46]. This is particularly concerning given the young age of participants in our study, and individuals who had four or more categories of ACEs are 4-12 times more likely to engage in health-risk behaviours (e.g., substance use, alcohol dependence, suicidality) and have chronic health problems later in life [14]. Further, we found a significant positive association between age and the total ACEs scores. This is unsurprising given that older children would have more time to be exposed to life adversity, thus highlighting the importance of early identification of ACEs and implementing appropriate interventions to prevent re-exposure of ACEs.

Numerous studies have documented the long-term repercussions (e.g., intergenerational trauma, disconnection from family and culture) of colonisation in Aboriginal communities [12, 47]. While Aboriginal participants with FASD were disproportionately represented in the current study, cultural background was not significantly associated with the total ACEs scores. This suggests other risk factors may explain the high rates of ACEs in this sample. Indeed, May and Gossage highlighted that children with FASD are often born into less stable families where parents are likely to be alcohol/substance users themselves [6]. Furthermore, many children with FASD display a range of challenging behaviours (e.g., lack of impulse control, immature social development, inability to predict consequences) as a result of underlying brain pathology [48]. Children with FASD may have sensory processing differences that can lead to them becoming easily upset/irritated by environmental triggers and having difficulties with emotional regulation and social pragmatics [49]. The challenging behaviours that children with FASD manifest could potentially increase the risk of them being exposed to ACEs (e.g., domestic violence, physical, emotional abuse) and pose difficulties for parents, some of whom will have experienced ACEs themselves and find parenting a child with a range of deficits difficult [48]. Most importantly, our findings highlight the importance of prevention/intervention, early screening and diagnosis of FASD in at-risk children to reduce the negative effects of ACEs and health risk behaviour.

### ACEs, Child Protection and Justice System Involvement

The proportion of individuals involved with the child protection system was much higher (70% in our sample vs. 56%) than a vulnerable/disadvantaged paediatric population from South Western Sydney [39]. Notably, higher total ACEs scores were associated with child protection system involvement in our study, with almost 50% of this subgroup having had four or more ACEs documented. Specifically, we found drinking/substance misuse at home, emotional neglect and physical neglect were strongly positively associated with child protection involvement. This is unsurprising given substance/alcohol misuse may compromise the parents’ ability to consistently provide a stable home environment and respond to the child’s emotional and physical needs [50]. Financial difficulties that arise from substance misuse may further compound the systemic issues faced by the family [50]. For parents with an intergenerational history of child protection involvement, drinking/substance misuse may be a maladaptive way to cope with their own traumatic experience of being removed from their families [51]. The issues that lead to children needing out-of-home care are often multifaceted and thus require a multiagency response that involves working with the families to build on parenting skills, strengthening support networks and assisting parents in getting help to address mental health problems (including intergenerational trauma) and substance/alcohol use challenges.

Interestingly, ACEs such as sexual abuse were not associated with child protection system involvement in this study even though sexual abuse, unlike other types of abuse, falls under the mandatory reporting legislation in WA [52]. A recent royal commission into violence, abuse, neglect and exploitation of people with disability shows that children with disability face additional barriers to disclosing abuse compared to neurotypical children [53]. For example, it is likely that underlying neurocognitive deficits (e.g., low intellectual functioning, poor expressive/receptive language skills) experienced by children with FASD may affect their abilities to comprehend the situation and report the abuse incident to relevant authorities. Unfortunately, this information was not collected in the study, so a formal relationship cannot be defined. Nevertheless, these findings have critical relevance for ACEs prevention in this clinical population and highlight the importance for child protection workers to routinely screen for ACEs in children with FASD.

Similar to previous research, almost half of the sample (40%) had been in contact with the justice system [54]. It has been proposed that the early life adversities and neurocognitive deficits (e.g., poor impulse control, lack of consequential thinking) associated with FASD can increase susceptibility to victimisation and involvement with the justice system [18]. In this study, exposure to domestic violence was positively associated with justice system involvement. This finding is consistent with a large body of research that demonstrated the relationship between domestic violence in childhood and antisocial behaviour in adolescents [55, 56]. It was highlighted in past studies that children who are exposed to domestic violence are more likely to suffer from mental health problems, struggle with forming secure attachments, be homeless, misuse alcohol/illicit substances; all of which are known risk factors for justice system involvement [57]. Given the far-reaching effects of domestic violence, early interventions are crucial to assist at-risk families.

### Associated Stressors

Of the overall sample, 43% disengaged from school. This finding is concerning as research has consistently established that students who disengage from school are more likely to engage in offending behaviour due to poor emotional connections with peers/teachers and having more time/energy available for illegal activities [58]. These findings emphasise the importance of early FASD screening at schools to identify their learning needs better and promote school engagement. Most importantly, those with FASD tend to have fewer protective factors due to their complex backgrounds and social vulnerability [59]. This study highlights the importance of FASD prevention programs and interventions that reduce the risk of children/young people experiencing criminogenic factors such as homelessness [60], bullying [61] and exposure to early life ACEs [62] to break the cycle of recidivism.

### Comorbidities

It is apparent that FASD is a risk factor for many comorbidities because of the physical consequences associated with PAE [20]. This interaction is further compounded by exposure to ACEs which is linked to increased rates of comorbid conditions [11]. We found higher ACEs scores were associated with more comorbidities, consistent with findings from previous research [11, 24]. For specific conditions such as attachment disorder, PTSD and substance use disorder, those with FASD also experienced more ACEs. This is unsurprising given past studies show higher ACEs scores predispose to the onset of PTSD and elevate the risk of illicit substance use [63].

Interestingly, comorbid conditions such as ID were not associated with increased ACEs scores, similar to findings from [24]. It may be that those with FASD and ID have better access to services and were protected against exposure to ACEs, though we did not examine this in our study. Furthermore, unlike PTSD, ID has strong prenatal/perinatal etiologies (e..g., genetic syndromes, brain malformations, neonatal encephalopathy) and may not necessarily be related to traumatic experiences; thus, this may also explain why ID was not associated with increased ACEs scores in our study [64]. Nevertheless, the high rates of neurodevelopmental/mental health comorbidities in this clinical population not only place a great demand on healthcare systems but also on parents/carers who are supporting a child with FASD, especially for those from disadvantaged backgrounds [65, 66]. Therefore, it is essential to ensure that caregivers are supported by specialised culturally secure and competent caseworkers who understand FASD and communicate well between medical/educational and community services.

### Strengths, Limitations, and Future Research

This research is valuable given children with FASD are at a heightened risk of experiencing ACEs over their lifespan. Understanding the nature and frequency of ACEs and other stressors in this clinical population is important as it can help inform FASD/ACEs prevention programs, interventions and the development of government policies [22]. A methodological strength of this study is the systematic and consistent assessments using internationally accepted FASD diagnostic criteria and the unique insight into the frequency and type of ACEs in Australian individuals with FASD.

This study has several limitations. Firstly, as ACEs data were retrospectively coded against the standardised ACEs questionnaire by the researcher rather than asking the caregivers directly thus, the number of ACEs may be underestimated. However, this was mitigated to a certain extent as ACEs data were collected from various sources, including allied health, medical, educational and diagnostic reports. Secondly, given the disproportionately high number of Aboriginal participants in this study when compared with the general population, the use of a traditional ACEs questionnaire may not accurately or adequately capture life adversities in this population as it did not consider, for example, the effects of colonisation and systemic racism [67]. This highlights the importance of working with Aboriginal communities to co-develop a culturally appropriate tool to better capture ACEs in this high-risk population. Due to the unique sociodemographic composition of our clinic sample (i.e., high-risk group with child protection/justice background), our findings may not be representative of the Australian FASD population as a whole. However, our results may be transferable to similar disadvantaged settings such as children in detention/child protection. There is also a male bias in our sample. This may be due to the high number of referrals Patches received from the justice system, where males are overrepresented, and the likelihood that males may be more vulnerable to the effects of PAE [68]. Nevertheless, investigations of ACEs in this clinical population should be followed-up by studies with a more equivalent number of male and female participants if possible.

Our study did not collect data on the number of placements and the timing of when the child was taken into care. It is possible that participants may have better outcomes and lower ACEs scores if they were placed into stable homes earlier and had fewer number of placements. Further investigation is required to examine the relationship between these variables and ACEs in an Australian FASD sample given past findings from a Canadian study among children/adolescents with PAE suggest that a higher number of placements is positively correlated with total ACEs scores [24]. Additionally, given the absence of a control group in this study, it will be important for future research to compare the life adversities of those with FASD versus those without to elucidate the unique effects of PAE on ACEs.

## Conclusions

Numerous studies have investigated the relationship between PAE and early life trauma. However, limited research has examined the ACEs profiles in individuals with FASD. Our study conducted a comprehensive examination of ACEs, associated stressors and comorbidities documented in children and youth with FASD in Australia. High rates of life adversity in this clinical population were associated with an increased number of comorbidities and negative outcomes, i.e., child protection/justice system engagement. Associated stressors (e.g., school disengagement, transiency, bullying, homelessness, traumatic brain injury) not captured by the ACEs questionnaire were also identified in this study. Overall, experiences of trauma are rarely an isolated event. Our results highlight a critical need for enhanced access to early diagnosis/services for children with FASD, particularly in higher-risk populations such as Aboriginal communities and those involved with child protection and/or the justice system to reduce the adverse impact of ACEs and the development of comorbid conditions. At a service provision level, it is crucial that clinicians/educators/child protection/justice workers routinely screen for, discuss, and provide psychoeducation around ACEs to promote better outcomes in vulnerable children (Fogliani, 2019).

## Supplementary Information

**Additional file 1.**

## Data Availability

The dataset used in the study is available from the corresponding author on reasonable request.

## Abbreviations

ACEs: Adverse childhood experiences
FASD: Fetal alcohol spectrum disorder
PAE: Prenatal alcohol exposure
ADHD: Attention-deficit hyperactivity disorder
PTSD: Post-traumatic stress disorder
ID: Intellectual disability
