# Supplementary material for: Adverse childhood experiences, associated stressors and comorbidities in children and youth with fetal alcohol spectrum disorder across the justice and child protection settings in Western Australia
Source: BMC Pediatr. 2022 Oct 10;22:587. doi: 10.1186/s12887-022-03654-y (PMC9549627; doi:10.1186/s12887-022-03654-y)
Supplement: Supplementary file 1 — Additional file 1. [file 12887_2022_3654_MOESM1_ESM.pdf]

Adverse Childhood Experience (ACE) Questionnaire
Finding your ACE Score ra hbr 10 24 06

While you were growing up, during your first 18 years of life:

1. Did a parent or other adult in the household **often** …
Swear at you, insult you, put you down, or humiliate you?
or
Act in a way that made you afraid that you might be physically hurt?
Yes No If yes enter 1 ________

2. Did a parent or other adult in the household **often** …
Push, grab, slap, or throw something at you?
or
Ever hit you so hard that you had marks or were injured?
Yes No If yes enter 1 ________

3. Did an adult or person at least 5 years older than you **ever**…
Touch or fondle you or have you touch their body in a sexual way?
or
Try to or actually have oral, anal, or vaginal sex with you?
Yes No If yes enter 1 ________

4. Did you **often** feel that …
No one in your family loved you or thought you were important or special?
or
Your family didn't look out for each other, feel close to each other, or support each other?
Yes No If yes enter 1 ________

5. Did you **often** feel that …
You didn't have enough to eat, had to wear dirty clothes, and had no one to protect you?
or
Your parents were too drunk or high to take care of you or take you to the doctor if you needed it?
Yes No If yes enter 1 ________

6. Were your parents **ever** separated or divorced?
Yes No If yes enter 1 ________

7. Was your mother or stepmother:
Often pushed, grabbed, slapped, or had something thrown at her?
or
Sometimes or often kicked, bitten, hit with a fist, or hit with something hard?
or
Ever repeatedly hit over at least a few minutes or threatened with a gun or knife?
Yes No If yes enter 1 ________

8. Did you live with anyone who was a problem drinker or alcoholic or who used street drugs?
Yes No If yes enter 1 ________

9. Was a household member depressed or mentally ill or did a household member attempt suicide?
Yes No If yes enter 1 ________

10. Did a household member go to prison?
Yes No If yes enter 1 ________

Now add up your "Yes" answers: _______ This is your ACE Score
